# Histopathologic Findings Following Experimental Equine Herpesvirus 1 Infection of Horses

**DOI:** 10.3389/fvets.2019.00059

**Published:** 2019-03-04

**Authors:** Carine L. Holz, Dodd G. Sledge, Matti Kiupel, Rahul K. Nelli, Lutz S. Goehring, Gisela Soboll Hussey

**Affiliations:** ^1^Department of Pathobiology and Diagnostic Investigation, College of Veterinary Medicine, Michigan State University, East Lansing, MI, United States; ^2^Veterinary Diagnostic Laboratory, College of Veterinary Medicine, Michigan State University, Lansing, MI, United States; ^3^Equine Hospital – Division of Medicine and Reproduction, Ludwig-Maximilians University, Munich, Germany

**Keywords:** EHV-1, EHV-4, histopathology, equine, EHM, ocular, testis

## Abstract

Histopathological differences in horses infected with equine herpesvirus type 1 (EHV-1) of differing neuropathogenic potential [wild-type (Ab4), polymerase mutant (Ab4 N752), EHV-1/4 gD mutant (Ab4 gD4)] were evaluated to examine the impact of viral factors on clinical disease, tissue tropism and pathology. Three of 8 Ab4 infected horses developed Equine Herpesvirus Myeloencephalopathy (EHM) requiring euthanasia of 2 horses on day 9 post-infection. None of the other horses showed neurologic signs and all remaining animals were sacrificed 10 weeks post-infection. EHM horses had lymphohistiocytic vasculitis and lymphocytic infiltrates in the lungs, spinal cord, endometrium and eyes. EHV-1 antigen was detected within the eyes and spinal cord. In 3/6 of the remaining Ab4 infected horses, 4/9 Ab4 N752 infected horses, and 8/8 Ab4 gD4 infected horses, choroiditis was observed. All males had interstitial lymphoplasmacytic and/or histiocytic orchitis and EHV-1 antigen was detected. In conclusion, only animals sacrificed due to EHM developed overt vasculitis in the CNS and the eye. Mild choroiditis persisted in many animals and appeared to be more common in Ab4 gD4 infected animals. Finally, we report infiltrates and changes in the reproductive organs of all males associated with EHV-1 antigen. While the exact significance of these changes is unclear, these findings raise concern for long-term effects on reproduction and prolonged shedding of virus through semen.

## Introduction

Equine herpesvirus type 1 (EHV-1) and 4 (EHV-4) are members of the subfamily *Alphaherpesvirinae* and are major pathogens of horses worldwide (1). Despite widespread vaccination, EHV-1 and EHV-4 infections remain a permanent risk. While the two viruses share a high degree of genetic and antigenic similarity, they differ significantly in host range and pathogenicity (2). Compared to EHV-4, which mainly infects horses and causes respiratory disease (2, 3), EHV-1 has a broader host range and can result in respiratory disorders, abortion, neonatal foal death, myeloencephalopathy and chorioretinopathy (4, 5). After initial infection, EHV-1 replicates in epithelial cells of the upper respiratory tract (6). Virus is then evident in lymphatic tissues, reaches regional lymph nodes, and infects mononuclear cells that ultimately reach the bloodstream, resulting in a cell-associated viremia (6). This cell-associated viremia allows for virus transfer and replication at the vascular endothelium of the target organs including the CNS, the pregnant uterus and the eye, and leads to vasculitis and ischemic thrombosis (7, 8) and subsequent abortion, neurological disorders, and ocular disease. Cell-associated viremia is not a consistent feature of EHV-4 infections and consequently, EHV-4 is only occasionally associated with abortion and extremely rarely with neurological disorders (2, 9–11).

One of the main problems in controlling equine herpesviruses infections is the virus' ability to establish latent infections from which virus can periodically reactivate, with the possibility of transmission to other individuals and the risk for EHM. Most alphaherpesviruses establish latency at the trigeminal ganglia, including bovine herpesvirus types 1 and 5, herpes simplex virus type 1 and type 2, Varicella zoster virus, and pseudorabies virus (12). Although neurotropism is a characteristic of alphaherpesviruses, it is not a constraint. For example, Marek's disease virus is lymphotropic (13). Latency for EHV-1 is established in the lymphoreticular system and in the trigeminal ganglia (14), where EHV-1 DNA can be detected (15). During latency, EHV-1 is predominantly, but not exclusively, recovered in lymphoid tissues draining the upper respiratory tract (16). In contrast to EHV-1, EHV-4 does not establish latency in nasal mucosa and peripheral lymphocytes, but only in the trigeminal ganglia (16–18).

Equine herpesvirus myeloencephalopathy (EHM) is one of the most severe manifestations of EHV-1 infection. The spinal cord is the part of the central nervous system that is most severely targeted during EHM, which results in ataxia and weakness, that can progress to recumbency requiring euthanasia (19). EHM may be suspected based on clinical findings, xanthochromia in the CNS and spinal cord histopathology characteristic of EHM (20). A definite diagnosis depends on a combination of a histopathologic examination of the brain and spinal cord in combination with serology, PCR and clinical presentation for confirmation of EHV-1 infection in a horse with suspected EHM. Vasculitis and thrombosis of small blood vessels in the spinal cord or brain are consistent histopathologic changes (21). However, spinal cord lesions can be easily missed on sectioning since they can be focal and only visible microscopically (20).

In addition, EHV-1 is the most important viral cause of equine abortion, both because it is relatively common and because of its potential for epizootic spread, particularly within naïve populations (22–24). Most of the abortions occur in the last third of pregnancy (22). Despite considerable research, the pathogenic mechanisms by which EHV-1 induces abortions and identification of host factors that are associated with susceptibility to EHV-1 abortion remain unexplained (25). Additionally, despite the fact that EHV-1 has a main tropism for the respiratory mucosa, the vaginal mucosa also supports replication (26). Previous reports also indicated that EHV-1 has been isolated from male genital organs and is shed with semen in sperm cells (27–30). Following intranasal infection of ponies with EHV-1 (Ab4) Tearle et al. described necrotizing vasculitis and thrombosis associated with viral replication in the vascular endothelial cells of the urogenital tract, as well as a decrease in the proportion of morphologically normal sperm (30), although the impact of EHV-1 infection on the sperm count has not been fully determined. It has been recently been proposed that venereal transmission of EHV-1 via the semen is possible, which had largely been ignored before (26). Venereal transmission of virus has been well documented for other alphaherpesviruses such as equine herpesvirus type 3 (31), bovine herpesvirus type 1 (32), and pseudorabies virus (33). In humans, HSV-2 can be detected in ejaculate samples and is associated with male infertility (34). HSV-2 can cause inflammatory disease of the genitourinary system of males, infects male sex cells, and is associated with poor sperm quality and decreased sperm concentration and motility. Finally, in a model of ascending urogenital HSV infection in mice, acute viral infection results in irreversible atrophy of the germinal epithelium, orchitis and infertility (35, 36), but studies in natural hosts of herpesviruses are lacking.

EHV-1 is also known to cause EHV-1-induced chorioretinopathy presenting as permanent focal or multifocal “shotgun” lesions of the chorioretina in a substantial proportion of infected horses (5, 37). This type of lesion is typically caused by vascular endothelial damage with subsequent ischemic injury to the chorioretina that results from direct infection of the vascular endothelium with EHV-1 (38). A recent study using experimental infection of horses with EHV-1 found that the ocular lesions generally develop between 4 weeks to 3 months after primary infection. Lesions can be focal or multifocal and unilateral or bilateral (5). Only in rare cases are the lesions diffuse affecting the entire eye and causing loss of vision. Because ocular lesions occur at the level of the chorioretina, which is hidden by the retinal pigmented epithelium (RPE), the ophthalmoscopic appearance of the lesions is not visible during the acute infection with EHV-1, but rather lesions only become visible once the RPE has been damaged (5). Thus, long-term studies are still needed to evaluate the evolution of those lesions in the horse.

There are primary and secondary sites of EHV-1 infection and the incidence of secondary disease is thought to depend on both viral and host factors (23, 39). Host factors affecting the incidence of EHM include age, sex and breed (39). For viral factors, a single nucleotide polymorphism in the viral DNA polymerase (ORF30, N752 replaced by D752) is significantly associated with an increase in neurovirulence of naturally occurring EHV-1 strains (40). In contrast, abortions are thought to be more commonly associated with the N752 genotype (26), although other factors are likely critical as well (41). The objectives of the current study were to evaluate lymphotropism/neurotropism/endotheliotropism of different EHV-1 viruses/mutants and a comprehensive evaluation of histopathological findings in horses infected with the neurotropic EHV-1 Ab4 strain (D752) and Ab4 mutants with differing neuropathogenic potential. For this a N752 mutant of the Ab4 strain was generated (Ab4 N752). In addition, an EHV-1/EHV-4 replacement mutant was made where glycoprotein D of EHV-1 was replaced with glycoprotein D of EHV-4 (Ab4 gD4) as a way of preventing/reducing viremia. Finally, we also wanted to determine the frequency of EHV-1-induced chorioretinopathy following experimental infection of horses with the different EHV-1 mutants *in vivo* by classical fundus photography and post-mortem histology.

## Materials and Methods

### Animals

Twenty-eight yearling horses of both sexes were used for this study. All animals were clinically healthy and exhibited virus neutralization titers < 4 for EHV-1 and < 40 for EHV-4 prior to experimental infection. Infections for each experimental group of horses were performed successively. Five horses served in both the control group and the Ab4 WT group. Animals were group-housed in a naturally ventilated barn and fed twice daily with a diet of hay and pelleted concentrate and *ad libitum* water throughout the experiment. All protocols were reviewed and approved by Michigan State University Institutional Animal Care and Use Committee.

### Viruses

The neuropathogenic EHV-1 strain Ab4 (D752 variant) used in this study was previously isolated from a quadriplegic mare (42). Additionally, two EHV-1 mutants of the Ab4 strain were used in this study. One was a mutant with decreased neuropathogenic potential (Ab4 N752) as described previously (43) and the second was a glycoprotein D (gD) mutant, where the gD from EHV-1 was replaced by gD from EHV-4 (Ab4 gD4) (44). Both viruses showed no significant differences in their *in vitro* growth curves but differed significantly in terms of clinical disease following experimental infection as described previously (44).

### Experimental Design and Infection

Animals were divided into four experimental groups for this study: Group 1 (*n* = 8) consisted of uninfected control horses (control group); group 2 (*n* = 8) horses were infected with Ab4 WT virus (Ab4 group); group 3 (*n* = 9) horses were infected with Ab4 N752 mutant virus (Ab4 N752 group); and group 4 (*n* = 8) horses were infected with Ab4 gD4 mutant virus (Ab4 gD4 group). Infections were performed on day 0 by intranasal instillation of 5 × 10^7^ plaque forming units (PFU) of the respective viruses in 10 ml of saline.

### Ocular Fundus Photography

Ocular fundus photography was performed in all horses 5 days prior infection, and 25, 45, and 70 days post-infection (p.i.). Ocular fundus photography in the uninfected control horses were performed at the beginning of the experiment, and 22, 64 and 85 days after the first ocular exam. For this purpose, horses were sedated with detomidine (0.02–0.04 mg/Kg, i.v.) and the pupil was dilated with 2 drops of tropicamide 1%, and phenylephrine 2% (Bausch&Lomb Inc., Tampa, FL, USA). Eye pictures were taken using an Optibrand ClearView™ Optical Imaging System (Dan Scott and Associates, Inc, Westerville, OH).

### Necropsy and Tissue Collection

Between 70 and 74 days p.i., horses from all infectious group were humanely euthanized by intravenous injection of sodium pentobarbital at 85.8 mg/Kg BWT. The exception to this were two horses in the Ab4 WT infectious group who were euthanized at day 9 p.i., as they developed serious signs of EHM such as reduced tail tone, hind limb ataxia, urinary incontinence, and recumbency. Respiratory (lung and trachea), lymphoid (tonsil, bronchial lymph node, retropharyngeal lymph node and mandibular lymph node), ocular (eyes), reproductive organs (testis, uterus, and ovaries), endocrine organs (adrenals and pituitary), nervous system (trigeminal ganglia, spinal cord, and brain), and other organs (spleen, heart, kidneys, and liver) tissues were collected from all EHV-1 infected horses. Samples were divided and snap frozen in liquid nitrogen for later DNA extraction or fixed in ice-cold 5% paraformaldehyde for 24 h before routine wax embedding and histologic processing.

### Histopathologic Examination

For histopathologic examination, 5 μm sections of formalin-fixed, paraffin-embedded portions of tissues collected during necropsies were examined using light microscopy. Evaluation of all organs included assessment for the presence or absence of necrosis, necrotizing vasculitis and inflammatory cell infiltrates, and the scoring of inflammatory infiltrates as mild, moderate, or marked, if present. For the testes, additional assessment was done for the degree of arteritis, presence of lymphofollicular aggregates, degree of interstitial inflammation, extension of inflammatory cells into seminiferous tubules, the number of interstitial pigment laden macrophages, the degree of tubular degeneration, and degree of atrophy of seminiferous tubules.

Immunohistochemistry for EHV-1 antigen was performed on representative sections of eye, spinal cord, and endometrium that contained regions of necrotizing vasculitis in horses that presented with clinical signs of EHM. In addition, immunohistochemistry was performed on representative testes and prostate in male horses from each infection group. For immunohistochemistry, 5 μm serial sections of formalin-fixed, paraffin-embedded tissue were labeled with a rabbit polyclonal anti-EHV-1 antibody (45). Immunohistochemistry was performed on the Discovery Ultra Automated Staining System (Ventana Medical Systems, Tucson, Arizona). Briefly, following automated deparaffinization and antigen retrieval for 8 min using protease 2 retrieval solution CC1 (Ventana Medical Systems), sections were incubated with a rabbit polyclonal EHV-1 antibody at a concentration of 1:6,000 for 1 hr. Labeling was detected using the polymer-based ultra-map universal alkaline phosphatase detection system with a red chromogen. Positive controls included tissues that had been shown to be positive for EHV-1 by PCR. For negative controls, the primary antibody was replaced with homologous non-immune serum.

### Analysis of Viral DNA From Testis Samples

A piece of the right and left testes were collected aseptically at the time of necropsy and immediately snap frozen into liquid nitrogen. Viral DNA was isolated from testes samples using the QIAamp DNA Blood Mini Kit, according to manufacturer's instructions (Qiagen Inc., Valencia, CA, USA). An amount of 25 mg of tissue was homogenized using TissueRuptor and sterile probes (Qiagen) in 1.5 mL safe-lock microcentrifuge tube. The presence of EHV-1 viral DNA in testes samples was determined using a previously described (44) real time PCR assay specific for gB gene of EHV-1 virus.

## Results

### Gross Necropsy Findings

For the two horses that were euthanized due to acute EHM, there was moderate hyperplasia of lymphoid tissues of the pharynx. The tracheal mucosa contained dozens of pinpoint petechiae and there were sharply demarcated, firm areas of dark red discoloration, 0.5–1 cm in diameter, randomly distributed throughout all portions of the lung. The pericardial sac contained approximately 500 ml of straw colored, transparent, serous fluid. In one of the two horses, there were ecchymoses over the C1-C2 spinal cord, and segmental areas within the C4-C7 cord were reddened. In this mare, the uterus contained an approximately 12 cm in length from crown to rump fetus. In the second horse, no gross lesions were seen in the spinal cord, but the urinary bladder contained moderate amounts of yellow sabulous material. In the remaining horses, who were euthanized at days 70–74 p.i., gross necropsy findings were unremarkable.

### Ocular Fundus Photography

Using fundus photography examination, we found that >60% of the horses in each group had shotgun eye lesions prior to infection and changes in numbers or quality of lesions were unremarkable at all time points following infection.

### Histopathological Findings

**Figure 1:**
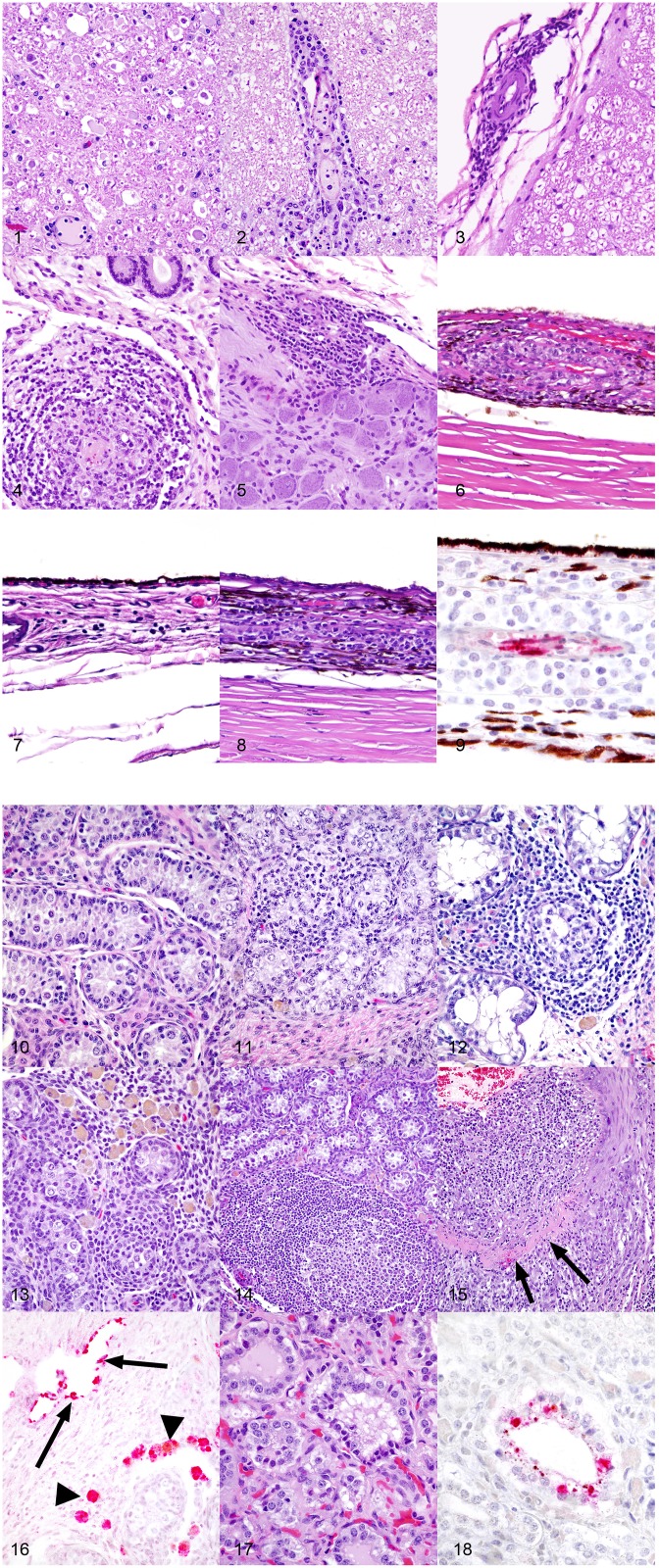
**(1)** Spinal cord, 1-year-old horse, 9 days post-equine herpesvirus-1 Ab4 WT infection. In this horse that had clinical signs of equine herpesvirus myelitis, there are regionally extensive areas of axonal degeneration including dilation of open myelin sheaths, axonal spheroids, and digestion chambers containing macrophages. Hematoxylin and eosin, 200x. **(2)** Spinal cord, 1-year-old horse, 9 days post-equine herpesvirus-1 Ab4 WT infection. There are thick cuffs of lymphocytes and histiocytes surrounding vessels. Hematoxylin and eosin, 200x. **(3)** Spinal cord, 1-year-old horse, 72 days post-equine herpesvirus-1 Ab4 WT infection. Mild infiltrates of lymphocytes, histiocytes, and plasma cells surround vessels of the meninges. Hematoxylin and eosin, 200x. **(4)** Endometrium, 1-year-old horse, 9 days post-equine herpesvirus-1 Ab4 WT infection. Dense infiltrates of lymphocytes and histiocytes surround and segmentally ablate the walls of endometrial vessels. Hematoxylin and eosin, 200x. **(5)** Trigeminal ganglion, 1-year-old horse, 72 days post-equine herpesvirus-1 Ab4 N752 infection. There are moderate perivascular and scattered interstitial infiltrates of lymphocytes, histiocytes and plasma cells. Hematoxylin and eosin, 200x. **(6)** Eye, 1-year-old horse, 9 days post-equine herpesvirus-1 Ab4 WT infection. Dense infiltrates of lymphocytes and histiocytes surround and segmentally ablate the walls of choroidal vessels. Hematoxylin and eosin, 200x. **(7)** Eye, 1-year-old horse, 72 days post-equine herpesvirus-1 Ab4 N752 infection. There are mild perivascular and scattered interstitial infiltrates of lymphocytes, histiocytes and plasma cells multifocally and randomly distributed throughout the choroid. Hematoxylin and eosin, 200x. **(8)** Eye, 1-year-old horse, 72 days post-equine herpesvirus-1 Ab4 gD4 infection. There are moderate perivascular and scattered interstitial infiltrates of lymphocytes, histiocytes and plasma cells multifocally and randomly distributed throughout the choroid. Hematoxylin and eosin, 200x. **(9)** Eye, 1-year-old horse, 9 days post-equine herpesvirus-1 Ab4 WT infection (serial section from the same horse represented in panel 6). There is strong stippled intracytoplasmic immunoreactivity for equine herpesvirus-1 (EHV-1) antigen within endothelial cells of vessels surrounded by dense inflammatory infiltrates. EHV-1 antigen immunohistochemistry, hematoxylin counterstain, 400x. **(10)** Testis, 1-year-old horse, 72 days post-equine herpesvirus-1 Ab4 WT infection. In histologically unremarkable regions of the testis, seminiferous tubules are lined by columnar Sertoli cells and few intermixed spermatogonia and are separated by plump polygonal interstitial (Leydig) cells and fibrous stroma. Hematoxylin and eosin, 200x. **(11)** Testis, 1-year-old horse, 72 days post-equine herpesvirus-1 Ab4 WT infection. Mild infiltrates of small lymphocytes and fewer histiocytes expand the interstitium and multifocally extend into and efface seminiferous tubules. There are rare histiocytes within the interstitium laden with pigment presumed to be lipofuscin. Hematoxylin and eosin, 200x. **(12)** Testis, 1-year-old horse, 72 days post-equine herpesvirus-1 Ab4 N752 infection. Moderate infiltrates of small lymphocytes and fewer histiocytes expand the interstitium and multifocally extend into and efface seminiferous tubules. There are rare histiocytes within the interstitium laden with pigment presumed to be lipofuscin. Hematoxylin and eosin, 200x. **(13)** Testis, 1-year-old horse, 72 days post-equine herpesvirus-1 Ab4 gD4 infection. Moderate infiltrates of small lymphocytes and fewer histiocytes expand the interstitium and multifocally extend into and efface seminiferous tubules. There are large numbers of histiocytes within the interstitium laden with pigment presumed to be lipofuscin. Hematoxylin and eosin, 200x. **(14)** Testis, 1-year-old horse, 72 days post-equine herpesvirus-1 Ab4 gD4 infection. Moderate infiltrates of small lymphocytes and fewer histiocytes, and few large secondary lymphoid follicles expand the interstitium. There are low numbers of histiocytes within the interstitium laden with pigment presumed to be lipofuscin. Hematoxylin and eosin, 100x. **(15)** Testis, 1-year-old horse, 72 days post-equine herpesvirus-1 Ab4 N752 infection. The wall of a muscular arteriole is segmentally ablated by dense expansile infiltrates of lymphocytes, histiocytes, and fewer neutrophils. In segmental regions, the smooth muscle of the tunica muscularis is replaced by a band of dense eosinophilic fibrin and necrotic debris. Hematoxylin and eosin, 100x. **(16)** Testis, 1-year-old horse, 72 days post-equine herpesvirus-1 Ab4 gD4 infection. There is strong intracytoplasmic immunoreactivity (red) for equine herpesvirus-1 (EHV-1) antigen in endothelial cells lining thin walled vessels and scattered macrophages within the interstitium. EHV-1 antigen immunohistochemistry, alkaline phosphatase red, hematoxylin counterstain, 200x. **(17)** Prostate, 1-year-old horse, 72 days post-equine herpesvirus-1 Ab4 gD4 infection. Few epithelial cells lining prostatic tubules are shrunken and hypereosinophilic and have pyknotic nuclei. Hematoxylin and eosin, 400x. **(18)** Prostate, 1-year-old horse, 72 days post-equine herpesvirus-1 Ab4 gD4 infection. There is strong stippled intracytoplasmic immunoreactivity (red) for equine herpesvirus-1 (EHV-1) antigen in scattered degenerate prostatic epithelial cells. EHV-1 antigen immunohistochemistry, alkaline phosphatase red, hematoxylin counterstain, 400x.

In horses infected with Ab4 WT that had clinical signs of EHM and were sacrificed at day 9 p.i., there were regions of overt vasculitis as evidenced by obscurement of vessel walls by necrotic debris and varying numbers of histiocytes in portions of the spinal cord, choroid of the eye, and endometrium (Tables 1, 2). These 2 EHM horses had regionally extensive areas of axonal degeneration including dilation of open myelin sheaths, axonal spheroids, and digestion chambers containing macrophages (Figure 1-1) and thick cuffs of lymphocytes and histiocytes surrounding vessels (Figure 1-2). Myelitis was rare in all horses that did not have clinical EHM with 1/5 horses infected with Ab4, and 1/9 horses infected with Ab4 N752 having mild perivascular infiltrates of lymphocytes and plasma cells within examined sections of spinal cord (Figure 1-3, Table 1). Of the 10 female horses without clinical signs of EHM, only one horse infected with Ab4 N752 had mild perivascular and scattered interstitial lymphohistiocytic infiltrates in the endometrium of the uterus. Some endometrial vessels had dense infiltrates of lymphocytes and histiocytes surrounding and segmentally ablating the wall (Figure 1-4, Table 1). All animals had mild lymphohistiocytic infiltrates scattered throughout the trigeminal ganglia (Figure 1-5, Table 1).

**Table 1:** Histopathological findings in the spinal cord, trigeminal ganglia, and uteri following infection with EHV-1 and EHV-1 mutants.

**Finding/group**	**Ab4 EHM**	**Ab4**	**Ab4 gD4**	**Ab4 N752**
Myelitis	2/2^*^	1/5	0/8	1/9
Trigeminal ganglioneuritis	Not examined	6/6	8/8	8/8
Endometritis	2/2^*^	0/3	0/2	1/6

* *Lesions in the spinal cord and endometrium of Ab4 WT infected horses with clinical signs of equine herpes myelitis included overt vasculitis. Inflammation in all tissues from horses without clinical signs of EHM was limited to mild perivascular to scattered interstitial infiltrates of lymphocytes and histiocytes*.

**Table 2:** Ocular findings following infection with EHV-1 and EHV-1 mutants.

**Experimental group**	**Ab4 EHM**	**Ab4**	**Ab4 N752**	**Ab4 gD4**
Necrotizing vasculitis	2/3	0/12	0/18	0/15
**DEGREE OF INTERSTITIAL CHOROIDITIS**				
None	0/3	6/12	6/18	0/15
Mild	1/3	6/12	12/18	12/15
Moderate	2/3	0/12	0/18	3/15

In addition to the necrotizing vasculitis in the choroid of the eyes seen in horses with clinical signs of EHM (Figure 1-6), examined eyes from animals in each experimental group had mild to moderate perivascular and interstitial infiltrates of lymphocytes and histiocytes (Figure 1-7, 8, Table 2). In horses infected with Ab4 and Ab4 N752 that did not have signs of EHM, there were no inflammatory infiltrates in a significant proportion of examined eyes (50 and 33.3%, respectively) and the remainder of eyes only had mild choroiditis (Figure 1-7, Table 2). Eyes from all horses infected with Ab4 gD4 had choroiditis and lymphohistiocytic infiltrates were moderate in 3/15 eyes (Figure 1-8, Table 2).

All males were sacrificed between days 70 and 74 p.i., and none of them exhibited clinical signs of EHM. While portions of testes were microscopically normal in all male horses (Figure 1-10), testes of all males contained some degree of perivascular to scattered interstitial lymphohistiocytic infiltrates, which often multifocally extended into and variably obscured the lining of seminiferous tubules (Figure 1-11–15, Table 3). In addition, 1/3 Ab4 and 2/6 Ab4 gD4 infected horses also had discrete lymphofollicluar structures within the interstitium of the testes (Figure 1-14). Tubular degeneration and atrophy in the absence of infiltrates of lymphocytes into seminiferous tubules was rare, respectively only being recognized in 1/4 Ab4 infected and 1/6 Ab4 gD4 infected, and 2/4 AB4 N752 infected horses (Table 3). The interstitium between seminiferous tubules of all infected horses contained dense infiltrates of large, foamy macrophages that often contained intracytoplasmic yellow to brown, lacy to finely granular pigment suggestive of lipofuscin (Figure 1-11–13). Such interstitial infiltrates of pigment laden macrophages were marked in all Ab4 and Ab4 gD4 infected males, but only seen in 1/4 Ab4 N752 infected male (Table 3). There was some degree of arteritis in the fascia surrounding the testes or fibrous interstitium within the testes of at least one horse from each group, and arteritis was marked in 1/4 Ab4 N752 infected horse (Table 3). Arteritis was characterized by variable infiltrates of lymphocytes and histiocytes into the tunica media and muscularis and/or segmental necrosis of the tunica muscluaris with variable replacement by fibrin (Figure 1-15). There were no obvious lesions in the ovaries of the mares and changes observed in the ovaries could not be distinguished from physiological changes in this age group (data not shown).

**Table 3:** Testicular findings following infection with EHV-1 and EHV-1 mutants.

	**Ab4 EHM**	**Ab4**	**Ab4 N752**	**AB4 gD4**
**ARTERITIS**				
	None	2/3	2/4	4/6
	Mild	1/3	1/4	2/6
	Marked	0/3	1/4	0/6
**LYMPHOFOLLICULAR AGGREGATES**				
	None	2/3	4/4	4/6
	Mild	1/3	0/4	0/6
	Marked	0/3	0/4	2/6
**INTERSTITIAL INFLAMMATION**				
	None	0/3	0/4	0/6
	Mild	3/3	4/4	5/6
	Marked	0/3	0/4	1/6
**TUBULAR INFILTRATE**				
	None	0/3	0/4	0/6
	Mild	3/3	3/4	6/6
	Marked	0/3	1/4	0/6
**PIGMENT LADEN MACROPHAGES**				
	None	0/3	0/4	0/6
	Mild	0/3	3/4	0/6
	Marked	3/3	1/4	6/6
**TUBULAR DEGENERATION**				
	None	3/3	3/4	5/6
	Mild	0/3	1/4	1/6
	Marked	0/3	0/4	0/6
**TUBULAR ATROPHY**				
	None	3/3	2/4	6/6
	Mild	0/3	2/4	0/6
	Marked	0/3	0/4	0/6

Using immunohistochemistry, EHV-1 antigen was detected within endothelial cells and histiocytes in regions of vasculitis in the spinal cord and eyes (Figure 1-9) of horses that had clinical signs of EHM. Viral antigen was also detected in the endothelium of thin walled vessels adjacent to regions of arteritis and in histiocytes filling the interstitium of the testes (Figure 1-16). While prostatic lesions were not appreciated on microscopic examination (Figure 1-17), there was rare immunoreactivity for EHV-1 antigen in the prostatic epithelium of one Ab4 N752 infected horse (Figure 1-18). When examining areas of IHC labeling on HE sections, few epithelial cells lining prostatic tubules were shrunken and hypereosinophilic and had pyknotic nuclei (Figure 1-17).

### Analysis of Viral DNA From Testis Samples

The quality of DNA extracted from these testicle samples was good (data not shown). Following real-time PCR analysis, the expression of β-actin was noticeably high, however EHV-1 gB gene expression was only detected at low levels (~35 cycles) in 50% of all males in the study.

## Discussion

This study represents the first report of gross anatomical and histological examination of horses euthanized 10 weeks after primary EHV-1 infection, when all clinical observations were within normal limits and horses had fully recovered from infection. Findings were compared with results in 2 horses from the same experiment, which were euthanized due to acute EHM at day 9 post-infection. This study also represents a comparison of tissues collected from horses infected with EHV-1 viruses of differing neuropathogenic potential.

As described previously, only horses infected with Ab4 showed signs of clinical EHM (3/8) (44). Pathologic findings in the 2 horses euthanized due to the severity of the symptoms on day 9 p.i. were consistent with lesion typically seen in horses euthanized following natural or experimental infection with EHV-1 (39, 41, 46, 47), and included endometritis, myelitis with axonal degeneration, perivascular cuffing and lymphocytic and histiocytic infiltrates as well as ocular necrotizing vasculitis and choroiditis with lymphohistiocytic infiltrates. Furthermore, EHV-1 antigen was detected by immunohistochemistry in eyes and spinal cord sections of the two mares with acute EHM. Induction of experimental EHM is highly variable and most studies use middle aged or older horses due to the known increased incidence of EHM in older horses (20, 41, 48, 49). Here we demonstrate that EHM can also be experimentally induced in yearling horses, but the incidence appears to be strain/virus dependent. This confirms previous studies in yearling horses where severe EHM was observed in individual horses following experimental challenge with Ab4 (41, 50).

Interestingly, significant histologic lesions were also observed in the majority of clinically healthy horses that were euthanized on day 70–74 p.i. in all three experimental groups. Ganglioneuritis with mild perivascular to scattered interstitial infiltrates of lymphocytes and histiocytes was present in sections of trigeminal ganglia of all horses. In addition, mild myelitis with mild perivascular to scattered interstitial infiltrates of lymphocytes and histiocytes was detected in one horse each from the Ab4 and Ab4 N752 infected groups. The persistence of histological findings in neural tissues and mononuclear/lymphocytic cell infiltrates long after clinical signs have disappeared, may be indicative of processes involved with the establishment, maintenance and reactivation from latency in lymphocytes and the trigeminal ganglia.

In addition to findings in the trigeminal ganglia and spinal cord, examined eyes from animals in all experimental groups had mild to moderate perivascular and interstitial infiltrates of lymphocytes and histiocytes at the time of euthanasia on day 70–74 p.i. Interestingly, 37.5% of horses infected with Ab4 and 33% of Ab4 N752 infected horses had no inflammatory infiltrates in the examined eyes and the remainder of eyes only had mild choroiditis. In contrast, eyes from all horses infected with Ab4 gD4 had choroiditis and lymphohistiocytic infiltrates were moderate in 3/15 eyes. This suggests that replacement of EHV-1 glycoprotein D (gD) with glycoprotein D from EHV-4 may have altered the endotheliotropism of the wild-type virus. Ocular fundus exams had been performed on all horses prior to euthanasia. A large number of horses exhibited shotgun lesions prior to experimental infection and no significant changes could be observed using fundus photography at any time prior to euthanasia. This is in contrast to previous studies in our laboratory where we could identify significant increases in ocular shotgun lesions using fundus photography in 50 and 70% of infected horses, respectively, by 3 months post-infection (5). However, in our previous studies, the incidence of lesions prior to challenge infection was very low and final fundus exams were taken later after infection (at 3 months p.i.), which may explain the lower incidence of new ocular lesions in the present study. The present findings indicate the limitation of ocular fundus photography in horses with pre-existing lesions, which may have been a result of exposure to EHV-1 shortly after birth, that would not have been detected by screening for VN antibody titers in the yearlings prior to the begin of the experiments. However, the current study supports previous studies and provides further evidence of the eye as a secondary site of EHV-1 manifestation and of vascular endothelial infection. A direct correlation between experimental EHV-1 infection and histologic ocular lesions in the present study is supported by the fact that EHV-1 antigen was detected by immunohistochemistry in horses with EHM. In addition, histologic lesions were present in eyes of horses that had no detectable shotgun lesions by fundus photography prior to experimental EHV-1 infection, suggesting that lesions developed as a result of challenge infection.

Finally, we report histologic lesions in the testes of all males and in female reproductive organs of individual mares. In addition to infiltrates of small lymphocytes and histiocytes that expand the interstitium and multifocally extend into, and efface seminiferous tubules, as well as histiocytes laden with pigment presumed to be lipofuscin, EHV-1 antigen was detected by immunohistochemistry in endothelial cells lining thin walled vessels of the testis, in scattered macrophages within the testicular interstitium, and within prostatic epithelium. Furthermore, 50% of all snap frozen testis samples were weakly positive for EHV-1 gB in 50% of all testes samples. Histologic lesions in the testis and epididymis including endothelial cell necrosis and lymphocytic infiltrates associated with EHV-1 antigen have previously been described in zebras (51), and in ponies following experimental infection with Ab4 (30, 52). In contrast to our study, lesions were detected during the acute phase of the infection, thus the present study is the first report demonstrating damage of the male reproductive organs of adolescent horses 10 weeks after primary infection. The study by Tearle et al. identified shedding of EHV-1 in semen and a significant decrease in the proportion of morphologically normal sperm as well as immature sperm at later collection time points (30). Presence of EHV-1 in semen has also been reported following natural outbreaks of EHV-1 (28, 29). Venereal transmission of EHV-1 via semen is possible (26), and has been well documented for other alphaherpesviruses such as equine herpesvirus type 3 (31), bovine herpesvirus type 1 (32), pseudorabies virus (33), and HSV-2 (34). However, the effects and relevance of EHV-1 on short term and long-term fertility in horses and the possibility of venereal transmission have not been formally investigated. While pathology and histopathology of EHV-1 abortions have been well investigated, the impact of EHV-1 infection on ovaries, particularly in young mares, remains to be further investigated.

In summary, we found that only animals sacrificed due to EHM had overt vasculitis in the CNS and the eye, and EHM occurred only following infection with Ab4. Mild choroiditis persisted in many animals and appeared to be more common in Ab4 gD4 infected animals. Finally, we report infiltrates and changes in the reproductive organs of all males and detection of EHV-1 antigen. While the exact significance of these changes is unclear, these findings raise concern for long-term effects on reproduction and prolonged shedding of virus through semen.

## Data Availability

All datasets generated for this study are included in the manuscript and/or the supplementary files.

## Author Contributions

GS is the senior author and designed the experiment, contributed to data collection and manuscript preparation and attracted funding for the study. DS and MK performed the necropsy and histopathology and helped with the manuscript preparation. CH helped with experimental design, sample collection, and manuscript preparation. RN performed the EHV-1 specific real time PCR on the testis. LG helped with the experimental design and sample collection.

### Conflict of Interest Statement

The authors declare that the research was conducted in the absence of any commercial or financial relationships that could be construed as a potential conflict of interest.
